# Seed-specific transcription factor HSFA9 links late embryogenesis and early photomorphogenesis

**DOI:** 10.1093/jxb/erx020

**Published:** 2017-02-16

**Authors:** Pilar Prieto-Dapena, Concepción Almoguera, José-María Personat, Francisco Merchan, Juan Jordano

**Affiliations:** 1 Departamento de Biotecnología Vegetal, Instituto de Recursos Naturales y Agrobiología de Sevilla, Consejo Superior de Investigaciones Científicas (CSIC), 41012 Seville, Spain; 2 Departamento de Microbiología y Parasitología, Facultad de Farmacia, Universidad de Sevilla, 41012 Seville, Spain

**Keywords:** Heat-shock factors, *Helianthus*, *annuus*, HSFA9, late embryogenesis, *Nicotiana tabacum*, photomorphogenesis, phytochromes, seed maturation, transcriptional control

## Abstract

HSFA9 is a seed-specific transcription factor that in sunflower (*Helianthus annuus*) is involved in desiccation tolerance and longevity. Here we show that the constitutive overexpression of HSFA9 in tobacco (*Nicotiana tabacum*) seedlings attenuated hypocotyl growth under darkness and accelerated the initial photosynthetic development. Plants overexpressing HSFA9 increased accumulation of carotenoids, chlorophyllide, and chlorophyll, and displayed earlier unfolding of the cotyledons. HSFA9 enhanced phytochrome-dependent light responses, as shown by an intensified hypocotyl length reduction after treatments with continuous far-red or red light. This observation indicated the involvement of at least two phytochromes: *PHYA* and *PHYB*. Reduced hypocotyl length under darkness did not depend on phytochrome photo-activation; this was inferred from the lack of effect observed using far-red light pulses applied before the dark treatment. HSFA9 increased the expression of genes that activate photomorphogenesis, including *PHYA*, *PHYB*, and *HY5.* HSFA9 might directly upregulate *PHYA* and indirectly affect *PHYB* transcription, as suggested by transient expression assays. Converse effects on gene expression, greening, and cotyledon unfolding were observed using a dominant-negative form of HSFA9, which was overexpressed under a seed-specific promoter. This work uncovers a novel transcriptional link, through HSFA9, between seed maturation and early photomorphogenesis. In all, our data suggest that HSFA9 enhances photomorphogenesis via early transcriptional effects that start in seeds under darkness.

## Highlight

HSFA9, a seed-specific factor, enhances the expression of light receptors and genes required for chlorophyll biosynthesis before germinating seeds are illuminated. HSFA9 subsequently increases phytochrome-mediated responses and stimulates seedling greening.

## Introduction

Plants use sunlight as the ultimate source of energy and also as one of the most important environmental cues. During seedling development immediately after seed germination, the absence or presence of light leads to two morphologically distinct developmental programmes of the plant. Seedlings that germinate under soil or litter adopt a dark-grown developmental programme called skotomorphogenesis, which stimulates the elongation of the embryonic stem, or hypocotyl, and represses cotyledon unfolding and chloroplast development. In contrast, when emerging from the ground or exposed to light, seedlings switch to a light-grown developmental programme called photomorphogenesis, which restrains hypocotyl growth and stimulates cotyledon unfolding and the development of photosynthetically active chloroplasts (Chen and Chory, 2011). The switch to photomorphogenesis is propelled by massive reprogramming of gene transcription (Leivar *et al.*, 2009). For example, in *Arabidopsis thaliana* up to one-third of nuclear-encoded genes are differentially expressed between wild-type seedlings grown in darkness and those grown under light (Ma *et al.*, 2001). After germinating seeds are exposed to light, photomorphogenesis is initiated by a suite of photoreceptors, including cryptochromes and phytochromes, which can jointly sense light from the blue to the far-red (FR) ends of the visible light spectrum (Kami *et al.*, 2010). Among these photoreceptors, *PHYA* and *PHYB* are essential for inducing photomorphogenesis (Franklin and Quail, 2010). Photomorphogenesis involves the coordinated transcription and translation of genes encoded by two genomes (the nuclear and chloroplastic). Seed embryos contain proplastids that develop into etioplasts during germination in the dark. Etioplasts are partially assembled plastids that complete their transformation into chloroplasts after young seedlings are exposed to light. Mature chloroplasts, which contain chlorophylls and the complete photosynthetic apparatus, can be formed only upon light-induced photomorphogenesis (Jarvis and López-Juez, 2013). A number of general regulators of photomorphogenesis have been identified, mainly in Arabidopsis (Jarvis and López-Juez, 2013; Leivar and Monte, 2014; Wu, 2014). These regulators include photomorphogenesis repressors, such as the *PIF* and *CONSTITUTIVE PHOTOMORPHOGENESIS 1* (*COP1*) genes, and activators, such as *ELONGATED HYPOCOTYL 5* (*HY5*) and *HYH* (Leivar and Monte, 2014; Wu, 2014).

In plants, the heat-shock response and some developmental processes are mainly controlled by a gene family of transcription factors (TFs) known as the heat-shock transcription factors (HSFs). Plants contain the highest number of HSFs among eukaryotes. The potential higher specialization of plant HSFs, compared with animal systems, is largely unexplored (reviewed by Scharf *et al.*, 2012). HSFs from few plant species other than Arabidopsis have been functionally analysed. These include HSFs that in zygotic embryos of sunflower (*Helianthus annuus* L.) activate a genetic programme that contributes to longevity, thermotolerance, and desiccation tolerance of seeds: the A9 programme. HSFA9 in sunflower and Arabidopsis are expressed only in seeds (Almoguera *et al.*, 2002; Kotak *et al.*, 2007); however, the equivalence of these two HSFA9 remains to be demonstrated. We note that a physiological characterization of the consequences of HSFA9 overexpression in Arabidopsis has not been reported yet; HSFA9 has been shown only to induce the accumulation of mRNAs from several heat-shock proteins (HSPs) under unstressed conditions in this model plant. Furthermore, mutation of HSFA9 did not have a phenotype in Arabidopsis (Kotak *et al.*, 2007). In contrast, loss of function of the A9 programme has been achieved in tobacco (*Nicotiana tabacum*) seeds, by using a repressor form of sunflower HSFA9 (A9-SRDX). A9-SRDX incorporates a potent, active repressor domain of 12 amino acids (designated SRDX), which contain an ERF-associated repression motif, fused to the C-terminus of HaHSFA9. Loss of function using A9-SRDX caused a significant reduction of seed longevity (Tejedor-Cano *et al.*, 2010; Carranco *et al.*, 2010). Functional analyses of plant HSFA9 would thus be currently limited to sunflower and similar dicot plants as tobacco, where precedent studies have determined a conserved regulation of the A9 programme—by HSF and non-HSF TFs— and the functional equivalence of their HSFA9 (Almoguera *et al.*, 2002; Díaz-Martín *et al.*, 2005; Carranco *et al.*, 2010; Tejedor-Cano *et al.*, 2014; and references therein). HSFA9 does not appear to be universally conserved in plants; monocots lack HSFA9 and some dicot plants, such as *Eucalyptus grandis*, have at least 17 closely related HSFA9 (Scharf *et al.*, 2012). This indicates functional diversification of HSFA9.

Skotomorphogenesis and photomorphogenesis are usually studied independently of zygotic embryogenesis, the developmental process that leads to the production of seeds. Here, we show that the seed-specific TF HSFA9 can accelerate the transition from skotomorphogenesis to photomorphogenesis. HSFA9 enhances gene expression relevant for photomorphogenesis in the dark, and also the initial light responses occurring after seed germination and seedling emergence from the soil. Some of the unexpected and complex effects caused by HSFA9 overexpression were confirmed by the observation of converse effects and gene expression changes after loss of function using A9-SRDX. Underlying part of the observed effects, HSFA9 would enhance light responses that depend at least on *PHYA* and *PHYB*. Such responses are critical to the transition from heterotrophic seedling growth from stored reserves to the biogenesis of photosynthetic active plastids, which allows autotrophic plant growth (Franklin and Quail, 2010; Ibarra *et al.*, 2013; Casal *et al.*, 2014, and references therein). In all, our results uncover a novel transcriptional link between seed maturation and early photomorphogenesis. The reported HSFA9 link would operate immediately upstream of the PHYA phytochrome photoreceptor, and have indirect effects on *PHYB*.

## Materials and methods

### Plant material and treatments

We used different pairs of homozygous transgenic and non-transgenic (NT, syngenic) tobacco (*N. tabacum* L.) sibling lines. The 35S:A9 (Prieto-Dapena *et al.*, 2008), DS10:A9 (Prieto-Dapena *et al.*, 2006), and DS10:A9-SRDX pairs (Tejedor-Cano *et al.*, 2010) have been previously reported. Each transgenic line corresponds to an independent, single transgene integration event in homozygosis. For each transgenic line a sibling NT line obtained from the parental, heterozygous transgenic line was used for the comparisons (as explained in Prieto-Dapena *et al.*, 2006).

Conditions for seed imbibition, germination, and seedling growth under a regular photoperiod of 16 h light/8 h dark have been previously described (Prieto-Dapena *et al.*, 2008). Imbibed seeds and seedlings were manipulated in darkness under dim (<0.05 µmol m^−2^ s^−1^) green safelight when required. White light treatments for the indicated times (6 or 16 h as indicated in the figures) were performed at the same light intensity, 100 µmol m^−2^ s^−1^, as in our conditions for growth under the regular photoperiod (Prieto-Dapena *et al.*, 2008). Continuous red light (660 nm) treatments at a fluence of 25 µmol m^−2^ s^−1^ or 35 µmol m^−2^ s^−1^ were performed at 23°C in a FITOCLIMA (model 1200 BIO) LED cabinet (Aralab, Spain). Continuous far-red light (730 nm) treatments at a fluence of 5 µmol m^−2^ s^−1^ were performed at the same temperature using a Percival Scientific (model E-30B, USA) LED cabinet. Average fluence rates were determined with a LI-COR (model LI250A, USA) radiometer. Hypocotyl length measurements were performed on scanned (≥600 dpi) images using the National Institutes of Health (NIH) Image software (Image J, National Institutes of Health, USA). Cotyledon expansion was estimated from photographs of the Petri dishes with the seedlings. Cotyledon unfolding was quantified by determining the percentage of seedlings with cotyledons that, after exposure to 16 h white light, expanded at least 120°.

### Suppression subtractive hybridization cloning

Suppression subtractive hybridization (SSH) was carried out using a SMART^TM^ PCR cDNA Synthesis kit and a PCR-Select Subtractive Hybridization kit (Clontech). All procedures were performed according to the manufacturer’s recommendations. The tester and driver cDNAs were synthesized using total RNA extracted by the LiCl procedure from 3-week-old NT_1_ and sibling 35S:A9_1_ seedlings, respectively. We used real-time quantitative PCR (RT-qPCR) to estimate the efficiency of subtraction by comparing the abundance of HaHSFA9 (A9) mRNA, and a non-differentially expressed gene, *L25*, before and after subtraction. This confirmed efficient subtraction (with a 200× increase in the relative abundance of A9). The PCR products derived from subtracted cDNA were cloned using the pGEM-T Easy T/A cloning vector system (Promega), also according to the manufacturer’s procedures. Ligation products were transformed into XL10-Gold^®^ ultra-competent cells (Stratagene).

### Real-time quantitative PCR

Total RNA was isolated from whole imbibed seeds or seedlings and further analysed by RT-qPCR. RT-qPCR was performed in a Roche LightCycler 480 and the SensiFast^TM^ SYBR^®^ No-ROX Kit (Bioline), using standard PCR conditions according to the manufacturer’s instructions. The cDNA used for RT-qPCR was prepared from 2 µg of total RNA (prepared with the LiCl method) using the Maxima First Strand cDNA Synthesis Kit for RT-qPCR (Thermo Scientific). We used about 20 ng of cDNA per RT-qPCR sample in 10 µl reactions. Parallel reactions were used to normalize the amount of template cDNA (Merchan *et al.*, 2007); normalized expression was calculated using the mean expression of three to four control transcripts. The specific primers for the four genes used for transcript abundance normalization [*Ntubc2*, *L25*, *EF-1α* (Schmidt and Delaney, 2010) and *NtPsbA*] are included in Supplementary Table S1 available at *JXB* online. Specificity was confirmed by analyses of the RT-qPCR profiles. Reproducibility of RT-qPCR was achieved by running technical duplicates, and by using two independent cDNA preparations. At least two biological replicates were performed per condition. Transcript levels of *COP1*, *HSP26*, *PsaG*, *PsbR*, *POR*, *HY5*, *PHYA1*, and *PHYB1* genes were analysed using the specific primers also listed in Supplementary Table S1.

### Photosynthetic pigment extraction and quantification

Individual samples collected from the same Petri dish (30–60 seedlings grown under darkness and then exposed to white light) were harvested on liquid nitrogen, and homogenized in 1 ml alkaline acetone (acetone to 0.1 N NH_4_OH in a volume ratio of 9:1). Chlorophyll was stepwise extracted with 1 vol, 1 vol, and 0.3 vol of 100% n-hexane. Thereafter, the unextracted chlorophyllide was determined by monitoring fluorescence emission at a wavelength of 675 nm using an excitation wavelength of 433 nm (Shalygo *et al.*, 2009). Total chlorophyll and carotenoid content was determined by UV–Vis spectroscopy (as in Almoguera *et al.*, 2015).

### Protein extraction and western blots

Western blots were performed essentially as described (Almoguera *et al.*, 2002), with the following modifications. Total seedling protein was extracted in boiling extraction buffer as described by Martín *et al*. (2016), except that 50 mM sodium bisulfite was used to substitute for β-mercaptoethanol in the extraction buffer. Imbibed seed proteins were extracted in 2× Laemmli buffer. The extracted protein, quantified by the Bradford assay (Biorad), was loaded on 8% SDS-PAGE gels. Amounts of 25 µg of total protein/lane were used for the seedling samples and 80 µg total protein/lane for the imbibed seed samples. PhyA proteins were detected using anti-phyA (Agrisera AS07 220) at 1/2000 dilution and goat anti-rabbit IgG (Agrisera AS09 602) at 1/25000 dilution. PhyB proteins were detected using the mAT5 antibody (López-Juez *et al.*, 1992) at 1/200 dilution and goat anti-mouse IgG (Calbiochem) at 1/5000 dilution. Incubations with the primary antibodies were performed overnight at 4°C.

### Laser scanning confocal microscopy

Chloroplast imaging using the fluorescence of chlorophyll was obtained using a Fluoview FV1000 microscope (Olympus) with a ×20 objective lens. Samples were excited with a multi-line Ar laser at 635 nm. Chloroplast autofluorescence emission (rendered in red) was recorded at 668 nm. To avoid fluorescence emission saturation in the recorded images, the following photomultiplier voltage settings were used: 35S:A9 and sibling NT samples, 450V; DS10:A9-SRDX and sibling NT samples, 550V. Ten seedlings were analysed per experiment and line. The average fluorescence intensity in arbitrary units (signal intensity) was quantified using the NIH Image software (Image J). Quantification used two 10 µm × 10 µm sections from each stacked image (a total of 34 –96 sections).

### Transient activation assays in sunflower leaves

A 1759 bp fragment of *NtPHYA1* (AWOK01600348.1) was PCR-amplified from *N. tabacum* genomic DNA using VELOCITY^TM^ DNA polymerase (Bioline) with the 5′-CAGGTCAAATCAATAGTGAAAAGG-3′ and the 5′-tgca*ccatgg*CGACTCAAATCTGAAATAGAACATC-3′ primers (the engineered *Nco*I site is italicized). The 1759 bp *NtPHYA1* fragment contains the major transcription initiation site (Adam *et al.*, 1997), transcribed 5′-flanking sequences including a conserved intron (577 bp), and 942 bp of non-transcribed promoter and upstream sequence. Another 757 bp fragment of *NtPHYB1* (AWOK01619430.1) was similarly amplified with the 5′-GCTTCTACAAAACTCAAAAACAC-3′ and 5′- tgca*ccatgg*CTCACAACTTTTCTTGGTTTAAC-3′ primers. This fragment contains 747 bp of sequences upstream of *NtPHYB1* translation initiation, which include the *NtPHYB1* transcription initiation sites (Adam *et al.*, 1997). The *NtPHYA1* and *NtPHYB1* fragments were cloned in the pSpark-I vector (Canvax) and, after verification of its DNA sequence, inserted as transcriptional fusions between the *Pst*I and *Nco*I sites (for *NtPHYA1),* or the *Not*I and *NcoI* sites (for *NtPHYB1*), of the pDR pSP101 (SK polylinker) plasmid (Díaz-Martín *et al.*, 2005). This originated, respectively, full-length PHYA:LUC and PHYB:LUC reporter constructs. The PHYA∆1:LUC and PHYA∆2:LUC reporter plasmids were constructed from PHYA:LUC by replacing the original *Pst*I-*Nco*I fragment (1803 bp) with shorter *Pst*I-*Nco*I fragments (907 or 844 bp, respectively). The mutant reporter construct was obtained from PHYA:LUC using the 5′-TCACTGCTTTtTAtTAAAGTCTCTCTCAC-3′ (substitutions indicated in lower case) and 5′-GTTGCCCAGTTGTTCTCTTTC-3′ primers with the Q5^®^ site-directed mutagenesis kit (New England Biolabs). The PHYB∆:LUC reporter plasmid was constructed by replacing the original *Not*I-*Nco*I fragment in the PHYB:LUC plasmid with a shorter *Pst*I-*Nco*I fragment (318 bp), which was obtained from the pSpark-I intermediate plasmid used for construction of PHYB:LUC. Bombardment of sunflower leaves was performed essentially as previously described (Díaz-Martín *et al.*, 2005). The amounts of plasmid DNA (per DNA precipitate, used for five shots) were: 5 µg of pBI221-HaHSFA9 (effector), 5 µg of reporter plasmid [PHYA:LUC, PHYA(m):LUC, PHYA∆1:LUC, PHYA∆2:LUC, PHYB:LUC, or PHYB∆:LUC], and 1 µg of pBI221-Rluc. The total amount of plasmid DNA was adjusted (if necessary) with pBI221 to 11 µg.

### Statistics

Detailed procedures for ANOVA analyses have been described previously (Prieto-Dapena *et al.*, 2006). We averaged the data for the different pairs of analogous sibling, transgenic, and NT lines. The statistical analysis of differences between the averaged data was consistent with results obtained when the differences were separately analysed for each sibling line pair (as in Almoguera *et al.*, 2015).

## Results

**Fig. 1.**
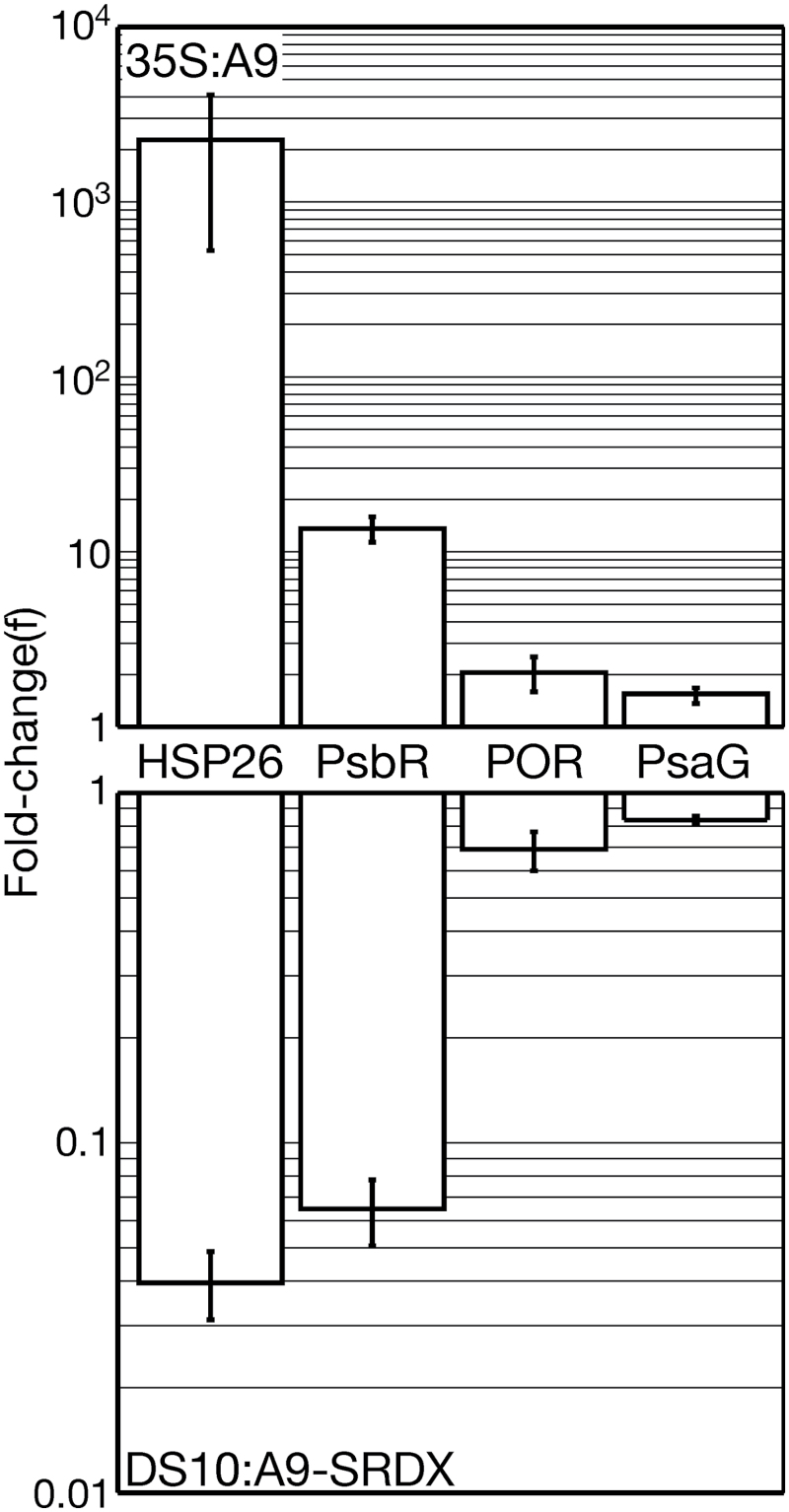
RT-qPCR analyses of transcripts from selected SSH-A9 clones. RT-qPCR fold-change induced by HSFA9 in gain-of-function lines (35S:A9) compared to non-transgenic (NT) siblings. Similar analyses for the loss-of-function DS10:A9-SRDX lines. The 35S:A9 and NT seedlings were grown under photoperiod light conditions for 3–4 weeks. The DS10:A9-SRDX and NT seeds were dark-imbibed for 24 h. Three different 35S:A9 or four DS10:A9-SRDX pairs of homozygous transgenic and NT sibling lines were analysed in at least two experiments. Error bars denote the SEM. The analysed transcripts include the plastidial sHSP *HSP26* (a previously known target of HSFA9), and mRNAs from three genes connected with the biogenesis of the photosynthetic apparatus (*PsbR*, *POR*, and *PsaG*, see the text for further details).

Our preceding work showed that the ectopic overexpression of HSFA9 in the transgenic tobacco 35S:A9 lines caused a remarkable protection of the photosynthetic apparatus from severe stress conditions (Almoguera *et al.*, 2012, 2015). The 35S:A9 seedlings also showed symptoms of an alteration of the photosynthetic apparatus under unstressed growth conditions, compared to NT sibling lines. These included higher PSII fluorescence (F_v_/F_m_), elevated accumulation of PSII proteins (PsbA and PsbP), and higher chlorophyll and carotenoid content (see Supplementary Fig. S1 at *JXB* online). These intriguing phenotypes were the first indication of new, unsuspected roles of HSFA9 in connection with the photosynthetic apparatus, in addition to the proposed protection from stress by HSPs encoded by the genes found to be activated by HSFA9 in the 35S:A9 plants (Prieto-Dapena *et al.*, 2008). To investigate an explanation for these phenotypes here, we used SSH (Merchan *et al.*, 2007) to identify genes, designated as SSH A9 clones, that might be directly or indirectly activated by HSFA9 in the 35S:A9 plants (199 ESTs from 168 unique genes, see Supplementary Table S2). Among the SSH A9 clones we noted a strikingly high proportion of genes (31%) involved in the photosynthetic apparatus. This included components of both PSI and PSII, the plastidial ATPase complex, the cytochrome b_6_f complex, and genes involved in the biogenesis of chloroplasts. RT-qPCR experiments confirmed that HSFA9 enhanced mRNA accumulation for genes such as *PsbR* and *PsaG*, with functions connected to the structure and assembly of photosynthetic membranes (Fig. 1, 35S:A9). HSFA9 also induced a *PORA*-like gene, which encodes a *NADPH:protochlorophyllide oxidoreductase* (POR, EC 1.3.1.33) catalysing the first light-dependent step of chlorophyll biosynthesis. The effects on the expression of genes relevant for photomorphogenesis, including the cloned *POR* gene, were corroborated by observing converse effects in dark-imbibed seeds from loss-of-function DS10:A9-SRDX lines (Tejedor-Cano *et al.*, 2010; Fig. 1). Here and in other similar experiments included in this report (see below), the use of the *DS10* promoter allowed efficient expression of HSFA9 in transgenic tobacco seeds (Prieto-Dapena *et al.*, 2006). Furthermore, the *DS10* promoter restricted HSFA9 overexpression to the time window in which the HSFA9 protein is present in sunflower (Almoguera *et al.*, 2002). POR is essential for the light-induced completion of the biogenesis of the photosynthetic membranes (Reinbothe *et al.*, 1999); therefore the results shown in Fig. 1 suggest that HSFA9 might favour photomorphogenesis.

### HSFA9 enhances photomorphogenesis and accelerates seedling greening

**Fig. 2.**
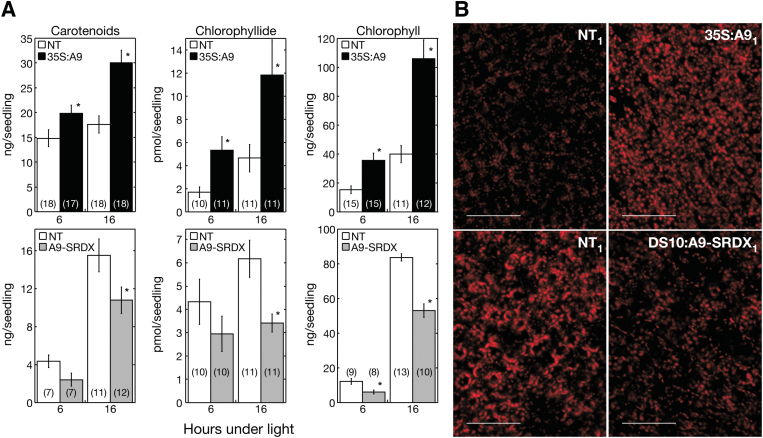
HSFA9 accelerates seedling greening. (**A**) Enhancement of carotenoid, chlorophyllide, and total chlorophyll content in the 35S:A9 lines and converse effects in the DS10:A9-SRDX lines. Top: 35S:A9 and non-transgenic (NT) sibling seeds were germinated and kept under darkness for 10 days. Bottom: DS10:A9-SRDX (A9-SRDX) and NT sibling seeds were germinated and kept under darkness for 4 days. After the respective dark germination treatments, plant seedlings were transferred to continuous white light conditions for 6 or 16 h, as indicated. Average photopigment content ± SEM was then determined in at least three experiments, performed with three different pairs of sibling lines. The asterisks denote significant statistical difference (*P* < 0.05). Numbers in brackets denote sample size (number of Petri dishes, each dish providing average measurements from 30–60 seedlings). (**B**) Enhanced intrinsic fluorescence of chloroplasts in the 35S:A9 seedlings and the converse effect observed in the DS10:A9-SRDX lines. Confocal microscopy images of the cotyledon chloroplasts of 35S:A9_1_ seedlings (top) or DS10:A9-SRDX_1_ seedlings (bottom). Average projections of the Z-stack reconstructions (10–18 slices, 2.45-µm thick each) are presented. Results obtained using the dark conditions in A, followed by exposure to white light for 16 h. Note that images were acquired under different conditions (for the 35S:A9 or DS10:A9-SRDX pairs). This only allows comparison with the respective sibling NT_1_ material shown on the left. Differences illustrated in the panels represent similar results consistently observed in four experiments performed with at least two sibling line pairs (in each case). Average fluorescence intensity was quantified for the different pairs of sibling lines. NT/35S:A9: 10.62 ± 2.25 and 19.20 ± 3.18, respectively. NT/ DS10:A9-SRDX: 17.36 ± 2.24 and 11.91 ± 0.55, respectively. Scale bars, 50 µm. This figure is available in colour at *JXB* online.

**Fig. 3.**
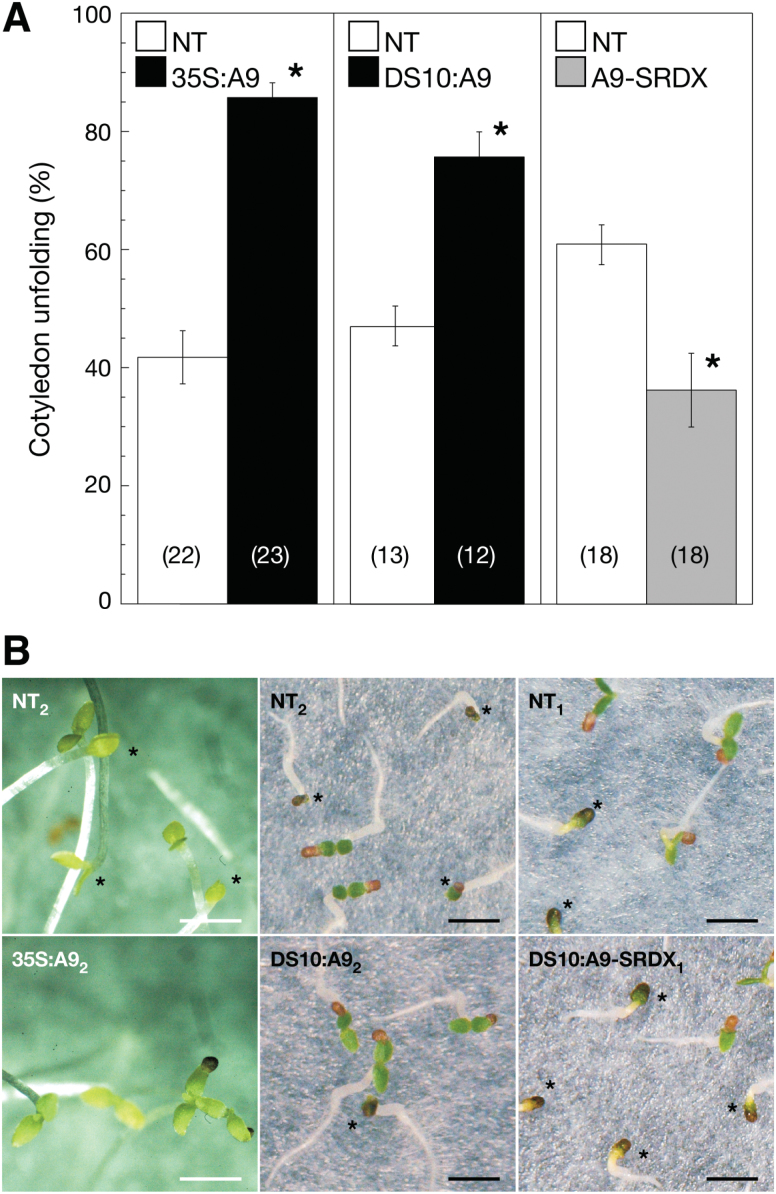
Enhanced cotyledon unfolding after exposure to light observed in the gain-of-function 35S:A9 and DS10:A9 seedlings, and the converse effect in the loss-of-function DS10:A9-SRDX seedlings. Cotyledon unfolding was scored after the etiolated seedlings (subjected to the same conditions as for the experiments in Fig. 2) were exposed to continuous white light for 16 h. (**A**) Average per cent values ± SEM of seedlings with fully expanded cotyledons. Results determined from at least two independent experiments performed with two to three pairs of lines with the 35S:A9, DS10:A9, or DS10:A9-SRDX (A9-SRDX) transgenes, and the corresponding sibling non-transgenic (NT) lines. Asterisks above bars denote statistically significant differences (*P* < 0.05). Numbers in brackets denote sample size (number of Petri dishes, each dish providing average measurements from 30–60 seedlings). (**B**) Representative results from the experiments in A are illustrated by photographs of the indicated pairs of sibling lines. Scale bars, 2 mm. The asterisks within the picture panels mark unexpanded cotyledons. This figure is available in colour at *JXB* online.

The results presented so far indicate that HSFA9 might enhance the initial photosynthetic development of seedlings. This suggestion was directly investigated by analysing greening induction of etiolated 35S:A9 seedlings exposed to light. For example, upon illumination for 6 or 16 h, both the chlorophyllide (produced in the step catalysed by POR) and the total chlorophyll content showed a significantly higher increase in the 35S:A9 seedlings compared to NT siblings. Total carotenoid content was similarly increased in the transgenic 35S:A9 seedlings (Fig. 2A, 35S:A9). Supplementary Table S3 summarizes the statistical data that support the observed differences. Confocal microscopy also showed that after the light treatments the intrinsic fluorescence of chloroplasts was higher in illuminated 35S:A9 seedlings than in sibling NT seedlings (Fig. 2B, 35S:A9; see also the average fluorescence data quantified in the legend for this figure), although chloroplast density did not differ between these plants (Supplementary Fig. S2). Remarkably, after exposure to light the 10-day-old 35S:A9 seedlings unfolded their cotyledons more efficiently than the NT siblings (Fig. 3, 35S:A9). Enhanced cotyledon unfolding by HSFA9 was also observed with the DS10:A9 lines at 4 days post-imbibition, a younger age within the time window in which the expression of endogenous HSFA9 still persists in sunflower (Almoguera *et al.*, 2002; Fig. 3, DS10:A9). Enhanced cotyledon unfolding is a physiological effect consistent with the observed enhanced greening of 35S:A9 seedlings in Fig. 2. To verify the effects of HSFA9 loss of function in connection with photomorphogenesis, DS10:A9-SRDX seedlings were exposed to light 4 days after seed imbibition. In line with the results for ectopic gain of function in 35S:A9 seedlings, the carotenoid, chlorophyllide, and total chlorophyll content decreased in the DS10:A9-SRDX seedlings compared to NT siblings (Fig. 2A). In addition, the fluorescence intensity observed after illumination was lower in the DS10:A9-SRDX seedlings compared to NT siblings (Fig. 2B). Finally, the illuminated DS10:A9-SRDX seedlings unfolded their cotyledons with significantly lower efficiency than NT siblings (Fig. 3, DS10:A9-SRDX). The results for the DS10:A9-SRDX seedlings in Figs 2 and 3, thus confirm by loss of function the effects of HSFA9 (on greening and cotyledon unfolding) in a specific time window—during early germination and seedling establishment—in which this embryonic TF is still present and active in sunflower (Almoguera *et al.*, 2002).

### HSFA9 enhances phytochrome responses

**Fig. 4.**
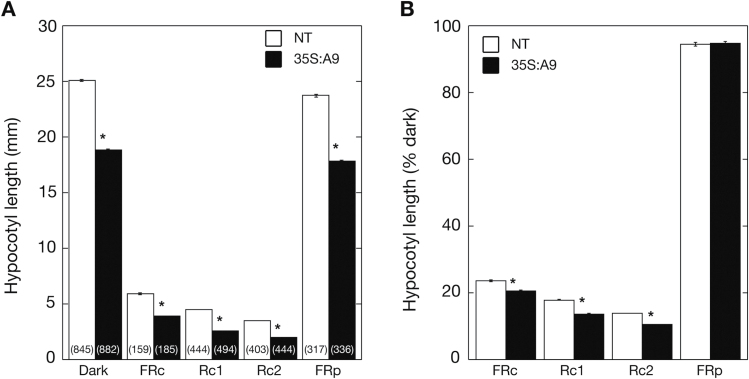
Hypocotyl growth inhibition of 35S:A9 seedlings: growth under darkness and responses to illumination with monochromatic light. (**A**) Hypocotyl length was measured in 10-day-old seedlings grown under darkness or under continuous monochromatic light at different fluence rates: 5 µmol m^−2^ s^−1^ far-red light (FRc), 25 µmol m^−2^ s^−1^ red light (Rc1), or 35 µmol m^−2^ s^−1^ red light (Rc2). A 20-min pulse of 15 µmol m^−2^ s^−1^ FR was applied just before the dark treatment in some experiments (FRp). (**B**) The measured hypocotyl lengths from panel A are represented as the percentage of length in light-grown (FRc or Rc) or FRp-treated seedlings in comparison to dark-grown seedlings. This eliminates the differences between non-transgenic (NT) and 35S:A9 seedlings observed without illumination and allows assaying of only the different (continuous or pulsed) light responses. Three pairs of 35S:A9/NT sibling lines were analysed in at least two independent experiments per light-treatment condition. Numbers in brackets represent the number of measurements specific for each light treatment; we also indicate the total number of dark-grown measurements used for normalization in B. Asterisks indicates the statistically significant differences mentioned in the text. Note that the FRp treatment did not have a significant effect (see Supplementary Table S3). Error bars denote the SEM.

Because HSFA9 could enhance seedling greening (Figs 2 and 3), and because we were seeking the mechanisms involved, our attention was directed to phytochrome light receptors. Among the phytochromes, *PHYA* and *PHYB* are perhaps the two photoreceptors with a higher contribution to the initial seedling greening (Franklin and Quail, 2010). We attempted to substantiate a possible phytochrome connection by analysing the effects of monochromatic light on hypocotyl length in seedlings. In transgenic tobacco the phenotypes affecting hypocotyl length could be studied only with the 35S:A9 lines. This is not feasible in the DS10:A9 seedlings, or after loss of function in the DS10:A9-SRDX seedlings, in contrast to the assessment of cotyledon unfolding (Fig. 3), because the hypocotyl lengths attained during seedling development in tobacco—and before the expression of HSFA9 driven by the *DS10* promoter is lost—are too short for quantification. Ten days after imbibition (and under darkness in sucrose-containing medium), the hypocotyls of 35S:A9 seedlings were significantly shorter than those of NT siblings (Fig. 4A). The presence of sucrose in the culture medium was found to increase the observed shortening; however, smaller but statistically significant differences between the hypocotyl lengths of 35S:A9 and NT seedlings were still observed in medium without sucrose (see Supplementary Fig. S3 at *JXB* online). Under continuous monochromatic light (FRc or Rc), the 35S:A9 seedlings also showed a statistically significant reduction of hypocotyl elongation compared to NT siblings. The observed changes occurred in addition to the reduction observed without the exposure to light in the presence of sucrose (Fig. 4B). These results revealed that HSFA9 enhanced light responses that are mainly mediated by *PHYA* or *PHYB*, respectively, under the FRc or Rc conditions used (see Nagatani *et al.*, 1993; Tepperman *et al.*, 2004).

The 35S:A9 lines also allowed us to investigate whether the effects of HSFA9 on hypocotyl length under darkness also depend on phytochrome photo-activation. The activating light could have come either from indirect exposure during seed development or from exposure to low-level ambient light during the manipulation necessary to imbibe the seeds. Careful treatments with a short pulse of FR light–applied immediately before the dark culture—were used to reverse the photo-activation of phytochromes. Hypocotyl lengths of the 35S:A9 and sibling NT seedlings were compared and the incidence of the FR pulse on the observed differences was assessed by normalizing lengths measured in treated seedlings with respect to those in seedlings not treated with the FR pulse. We found that the FR pulse did not change the differential hypocotyl length shortening observed in the 35S:A9 seedlings (Fig. 4B). Reduction of hypocotyl length under darkness by HSFA9 would not thus depend on pre-existing phytochrome photo-activation.

### HSFA9 enhances gene expression relevant for photomorphogenesis in imbibed seeds

**Fig. 5.**
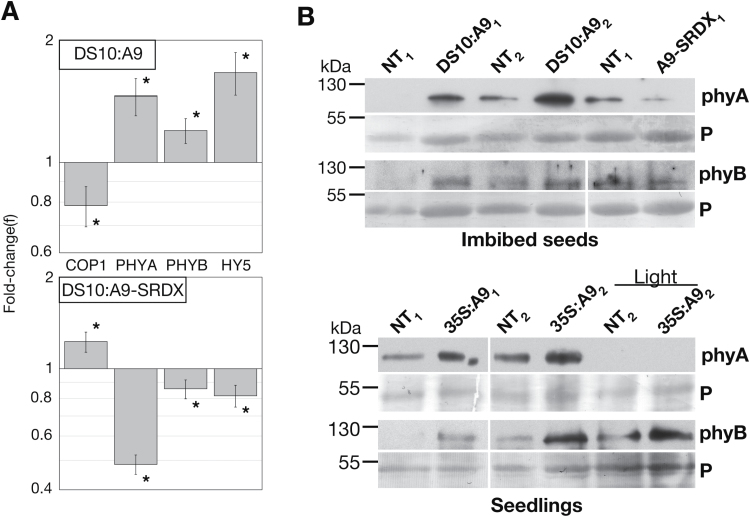
Effects of HSFA9 in seeds and seedlings before the first exposure to light. (**A**) Changes in transcript levels for *COP1*, *PHYA*, *PHYB*, and *HY5* in DS10:A9 seeds, and converse trend as determined by RT-qPCR, in seeds from the loss-of-function DS10:A9-SRDX lines. The seeds were imbibed under darkness for 24 h. Two to three different DS10:A9 or two to four DS10:A9-SRDX pairs of homozygous transgenic/non-transgenic (NT) sibling lines were analysed in at least two experiments. Error bars denote the SEM. The asterisks denote that all fold-changes were statistically significant (the depicted ratios were different from 1; see Supplementary Table S3). (**B**) Enhanced accumulation of phyA and phyB antibody-reacting proteins under darkness in imbibed seeds and seedlings. Top: seeds imbibed for 24 h, results depicted for the indicated sibling pairs of NT/DS10:A9 and NT/DS10:A9-SRDX (A9-SRDX) lines. Bottom: seedling results illustrated for two different sibling pairs of NT and 35S:A9 lines. The putative phyA band disappears (as expected) after illumination for 6 h with white light (Light). Equal loading of total protein in samples was verified with Ponceau S staining (P).

In dicot plants, the expression of HSFA9 is restricted to seed embryos (Almoguera *et al.*, 2002; Kotak *et al.*, 2007), and in sunflower the HSFA9 protein has been shown to disappear approximately 5 days after seed imbibition and germination. Subsequent heat- or drought-stress treatments fail to re-induce HSFA9 later in development (Almoguera *et al.*, 2002). These expression patterns suggest that HSFA9 could affect gene expression relevant for photomorphogenesis much earlier than in the stages analysed for 35S:A9 seedlings in the experiments presented so far. To investigate this, we studied molecular effects caused by HSFA9 using different sibling pairs of DS10:A9/NT lines under dark culture conditions (Prieto-Dapena *et al.*, 2006). In the DS10:A9 lines (as in the DS10:A9-SRDX loss-of-function lines), overexpression of HSFA9 occurs only in developing seeds. We would assume a similar localization for endogenous HSFA9 and for DS10-induced HSFA9: in both cases in embryos, and perhaps homogeneously distributed. This is inferred from the homogeneous localization of seed small HSPs (sHSPs) in sunflower seed embryos (Coca *et al.*, 1994) and from the effects for DS10:A9 on seed sHSPs and seed longevity (Prieto-Dapena *et al.*, 2006). After seed imbibition for 24 h under darkness, we used RT-qPCR to analyse the expression of *PHYA* and *PHYB*, as well as possible expression changes for a main repressor (*COP1*) and an activator (*HY5*) of photomorphogenesis (Deng *et al.*, 1991; Lau and Deng, 2012; Oyama *et al.*, 1997). Enhanced accumulation of the transcripts for *PHYA* and *HY5* was observed but, interestingly, transcript accumulation of the *COP1* repressor was slightly reduced (Fig. 5A; DS10:A9). In the case of *PHYB* transcripts, accumulation in seeds was enhanced, although at lower fold levels than for *PHYA* and *HY5*. All the mentioned changes, including the very mild effects on *PHYB* and *COP1* transcript accumulation, were statistically significant (Supplementary Table S3). Furthermore, all these effects of HSFA9 were confirmed by loss of function by the observation of the converse expression change in each case (Fig. 5A, DS10:A9-SRDX). Western blot analyses using specific antibodies (see Materials and methods) showed enhancement in the accumulation of putative phyA and phyB proteins in DS10:A9 seeds after dark imbibition for 24 h. Furthermore, the converse phyA and phyB protein accumulation changes were observed in imbibed DS10:A9-SRDX seeds (Fig. 5B; imbibed seeds panel). Additional immunoblots showed that, in 35S:A9 seedlings grown for 10 days under darkness, the accumulation level of a protein band that matched the expected mobility and light-sensitivity of phyA was enhanced, compared to NT siblings. In these 35S:A9 seedlings, the accumulation of putative phyB proteins was also enhanced before the first exposure to light (Fig. 5B; seedlings panel). We conclude that HSFA9 might enhance photomorphogenesis with early gene expression effects that start under darkness in seeds. The observed molecular and physiological effects appear to involve the upregulation of at least two different phytochrome light receptor genes: *PHYA* and *PHYB*.

### HSFA9 activates the NtPHYA1 promoter

**Fig. 6.**
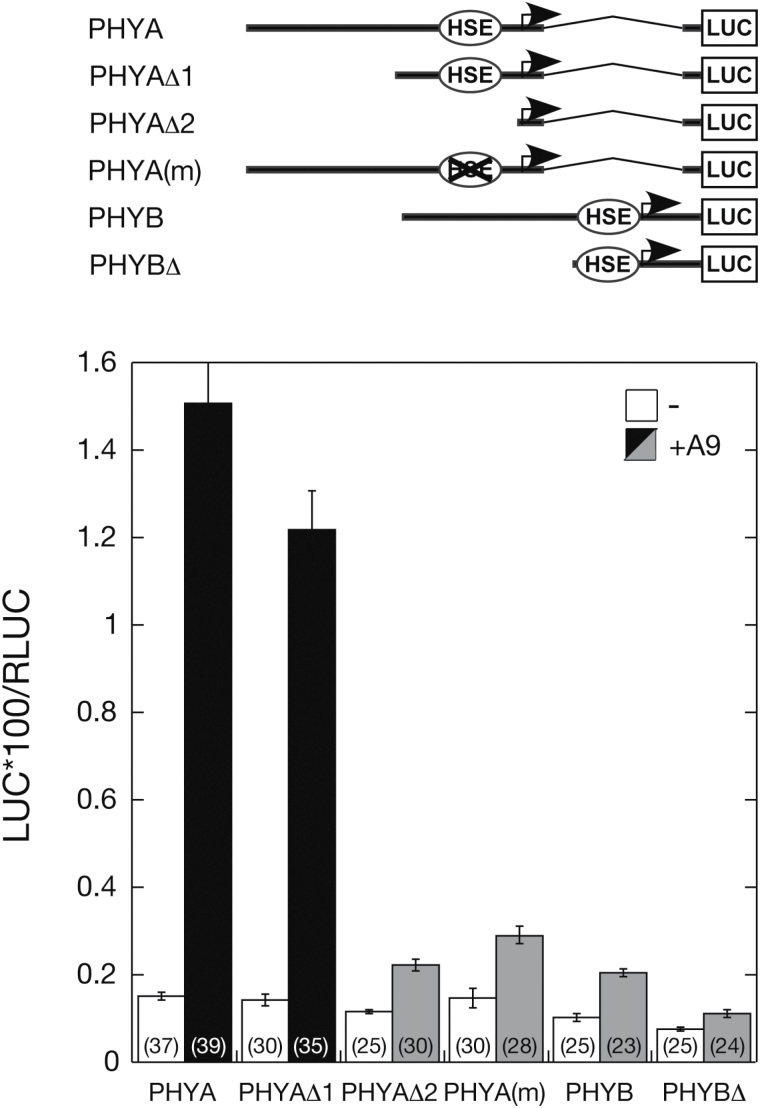
HSFA9 activates the *NtPHYA1* promoter. Top: cartoon showing the different reporter genes used in the transient expression assays (genes are not drawn to scale). HSE denotes putative imperfect *cis*-elements, including the one used by HSFA9 in *NtPHYA1*; this element was mutated in the PHYA(m) construct (see Supplementary Fig. S4). Bottom: transient expression assays. Numbers in parentheses show the number of replicates for each reporter/effector plasmid combination. The same bar shading indicates similar LUC reporter activity. Error bars denote the SEM.

To investigate a possible transcriptional link involving HSFA9 and the phytochromes upregulated by HSFA9 (Fig. 5), we used transient assays to analyse if HSFA9 activates the promoters of the genes analysed by RT-qPCR. The results in Fig. 6 showed a distinct transcriptional activation of the *NtPHYA1* promoter by HSFA9. Deletion analyses indicated that the observed activation requires the proximal 5′-flanking sequences that contain an imperfect heat-shock element (HSE) *cis*-element located between positions -55 and -67 (Fig. 6, compare the full-length PHYA construct with the PHYA∆1 and PHYA∆2 constructs). This *cis*-element is similar to the HSE found in the specific *sHSP* gene promoters that are activated by HSFA9 in seeds (Carranco *et al.*, 1999 and references therein). Two nucleotides at crucial positions within this HSE (see Supplementary Fig. S4 and Carranco *et al.*, 1999) were mutated in the context of the full-length PHYA:LUC construct. Mutation of these residues, which would only impair DNA-binding of HSFA9 and similar HSFs (Carranco *et al.*, 1999), sufficed to abolish the transcriptional activation of the *NtPHYA1* promoter by HSFA9 [Fig. 6, PHYA(m)]. These results suggest a direct transcriptional effect of HSFA9 on the *NtPHYA1* promoter through this proximal and imperfect HSE. In contrast, HSFA9 only activated the promoter of the *NtPHYB1* gene to levels similar to those found when using the PHYA∆2 or PHYA(m) constructs. Thus, this activation was only marginal (less than 2-fold), and it occurred in spite of the presence of putative HSEs, which in this case do not appear to be used by HSFA9 [Fig. 6, compare PHYB and PHYB∆ with PHYA∆2 or PHYA(m)]. Thus, HSFA9 might directly activate the *NtPHYA1* promoter, but the effect on the *NtPHYB1* promoter would be indirect.

## Discussion

### HSFA9 links late embryogenesis and early photomorphogenesis in sunflower and similar dicot plants

We propose that HSFA9—a seed-specific TF—mediates a transcriptional link between seed maturation and photomorphogenesis. We posit that HSFA9 contributes to restricting skotomorphogenesis and enhancing early photomorphogenesis, with it powering photomorphogenesis upstream of at least two phytochromes: *PHYA* and *PHYB*. The effects on *PHYB* are perhaps indirect, but HSFA9 might directly activate *PHYA,* as inferred from transient assays (Fig. 6). Very little is known about TFs that modulate phytochrome gene expression. Phytochrome promoter analyses have been performed only for monocot plants; only the GT-2 TF has been shown to activate *PHYA,* as determined by transient expression assays similar to those performed in the experiments shown in Fig. 6 (Dehesh *et al.*, 1990; Bruce and Quail, 1990; Ni *et al.*, 1996; Martínez-García and Quail, 1999). No similar dicot TFs have been functionally analysed; the described monocot TF would be involved in constitutive activation (or downregulation) of *PHYA* promoters, rather than in the developmental regulation here proposed for HSFA9. The effects of HSFA9 would start occurring in imbibed seeds early and in the dark, coincidently with the persistence of the seed-stored HSFA9 protein in sunflower (Almoguera *et al.*, 2002).

In the literature we found only a few precedents for the existence of regulatory links between embryogenesis and photomorphogenesis (or skotomorphogenesis). Only very recently it was found that LEAFY COTYLEDON1 (LEC1), a master regulator in embryogenesis and seed maturation, enhances skotomorphogenesis (Huang *et al.*, 2015). LEC1 acts as a co-activator of hypocotyl elongation-related genes during post-embryonic development of early seedlings under darkness (Huang *et al.*, 2015). Similar connections with photomorphogenesis have been analysed in less depth and the linking TFs remain largely unknown. For example, some studies have shown that gene expression during late embryogenesis affects chloroplast biogenesis in seedlings of Arabidopsis. In one case, oxidative cues provide the regulatory link through thylakoid-localized EXECUTER (EXE) proteins (Kim *et al.*, 2009). The EXE proteins indirectly affect nuclear transcription that is relevant for chloroplast biogenesis. Most likely this occurs through the transmission of oxidative cues imprinted on the EXE proteins by singlet-oxygen (Kim *et al.*, 2009) to nuclear-localized TFs that are still unidentified. Overexpression of ABSCISIC ACID INSENSITIVE3 (ABI3) leads to the preservation of chloroplasts in etiolated Arabidopsis plants. This suggests that ABI3, a master TF regulator of plant embryogenesis and seed maturation (To *et al.*, 2006), acts negatively on the transition from chloroplast to etioplast (Rohde *et al.*, 2000). We note that in Arabidopsis ABI3 activates HSFA9 (Kotak *et al.*, 2007), and that ABI3 is similarly networked to HSFA9 in other plants, such as *Medicago truncatula* (Verdier *et al.*, 2013).

Dicot plants other than sunflower, such as Arabidopsis or *M. truncatula*, have HSFA9 putative homologs that might be involved in a regulatory connection similar to the one reported here. However, we should stress that the functional equivalence of the different HSFA9 remains to be demonstrated. This is not a trivial task, and a direct translation of the implications of the results reported here to different dicot plants is not currently possible. Sunflower and tobacco are closely related dicot plant species (as both belong to the Asterid clade). In Asterid plants the A9 programme is under a complex, redundant regulation that involves additional HSF and non-HSF TFs (Almoguera *et al.*, 2002; Díaz-Martín *et al.*, 2005; Carranco *et al.*, 2010; Tejedor-Cano *et al.*, 2014; and references therein). This regulation might differ in divergent dicot species such as Arabidopsis (belonging to the Rosid clade); for example, HSFA4a, an additional sunflower HSF involved in the co-activation of the A9 programme, would have a similar function in tobacco, whereas Arabidopsis HSFA4a differs from sunflower HSFA4a (Tejedor-Cano *et al.*, 2014). These differences complicate the extension of our model to non-Asterid dicot plants. In addition, the lack of available mutants and the incomplete genetic characterization of phytochrome signalling in tobacco limited the depth of our study. Monocot plants lack HSFA9 (Scharf *et al.*, 2012). In this case, seed TFs other than HSFA9 and acting downstream of ABI3 (or downstream of a different master regulator of seed development) may still link embryogenesis and photomorphogenesis.

### HSFA9 affects gene expression relevant for photomorphogenesis before seeds and seedlings are exposed to light

The transcriptional effect of HSFA9 would impact the switch between skotomorphogenesis and photomorphogenesis very early (before mature seeds and young seedlings are exposed to light). This is supported by consistent results of both gain and loss of function obtained in seeds imbibed for 24 h under darkness (Fig. 5; see DS10:A9 and DS10:A9-SRDX, respectively). Under these conditions HSFA9 caused a very mild, but statistically significant, reduction of transcript accumulation of *COP1*, which is a gene that acts as a master repressor of photomorphogenesis (Deng *et al.*, 1991; Lau and Deng, 2012). These results suggest that the native HSFA9 might help to reduce the negative effects of COP1 in seeds germinating under darkness. In contrast, HSFA9 enhanced the expression of *HY5*, an activator TF with a key role in light signalling and early photomorphogenesis that under darkness is ubiquitinated and targeted for degradation by COP1 (Oyama *et al.*, 1997; Osterlund *et al.*, 2000). The enhanced transcript accumulation of *HY5* in dark-imbibed DS10:A9 seeds coincided with a similar effect on *PHYA* and *PHYB*, which in this case could be confirmed at the protein level (Fig. 5B). We propose that the early effects of HSFA9 contribute to photomorphogenesis and prepare dark-imbibed seeds for subsequent responses to light. Seed-stored HSFA9 (Almoguera *et al.*, 2002) could be involved in maintaining a described small pool of HY5 protein that is present before light exposure (Hardtke *et al.*, 2000). HSFA9 would also enhance the pool of seed-stored phyA and phyB proteins (Fig. 5B), perhaps as a result of both transcriptional and post-transcriptional effects. Indeed, the effects of HSFA9 on *PHYA* and *PHYB* were more obvious at the protein than at the transcript level (compare Fig. 5A with Fig. 5B). This suggests that effects such as enhanced translation (and/or decreased protein degradation) might amplify the modest transcript accumulation changes observed for these two phytochromes (and in particular for *PHYB*).

It is worth highlighting that the reduction of skotomorphogenesis by HSFA9 does not seem to require phytochrome photoreceptor activation. This is inferred from the effect of FR light pulses on the differential reduction of hypocotyl length in 35S:A9 seedlings grown under darkness. These pulses should have reverted any active phytochrome present before the seedling growth under complete darkness was analysed. Because the pulses did not affect this reduction (Fig. 4), HSFA9 most likely decreases hypocotyl growth under darkness by mechanisms that are independent of photo-activated phytochromes. We cannot rule out negative effects of HSFA9 on PIF factors, which are known to enhance hypocotyl growth under darkness (reviewed by Leivar and Monte, 2014). However, our results would exclude at least some mechanisms; for example, that HSFA9 enhances PIF degradation involving photo-activated phytochromes (reviewed by Leivar *et al.*, 2008), because the FR pulses would have reverted (at least in part) such an effect. We note that there is evidence in the literature for signals from imbibed seeds that modulate hypocotyl growth under darkness at the subsequent seedling stages in Arabidopsis. These trans-developmental signals are mediated by *PHYA* and *PHYB*, but similar to our results, do not involve photoactive phytochromes (Magliano and Casal, 2004). We also note that adding sucrose to the culture medium causes a strong reduction of hypocotyl elongation in Arabidopsis seedlings grown under illumination, but enhances elongation under darkness (Zhang *et al.*, 2010). Thus, the higher reduction of hypocotyl lengths, compared to NT siblings, observed in 35S:A9 seedlings grown under darkness in sucrose-containing medium [compared to what was observed in medium without sucrose (Supplementary Fig. S3)] would indicate an anticipated light response in the 35S:A9 seedlings.

### HSFA9 reinforces phytochrome signalling and accelerates seedling greening upon first exposure to light

The initial responses to light of seedlings are required for the continuation of photomorphogenic development after seedlings emerge from soil. Enhanced light responses, coupled to the earlier changes induced by HSFA9 in seeds under darkness, would therefore facilitate the light-induced completion of the biogenesis of the photosynthetic apparatus. This was indeed observed in the 35S:A9 seedlings; furthermore, it was also confirmed by loss of function, and within the expression window of native HSFA9 (Almoguera *et al.*, 2002), using the DS10:A9-SRDX seedlings (Fig. 2). In addition, enhanced cotyledon unfolding by HSFA9 was confirmed in the DS10:A9 seedlings within the same expression window (Fig. 3). Therefore, native HSFA9 would be able to enhance photomorphogenesis before its expression shuts off in very young seedlings (Almoguera *et al.*, 2002); constitutive overexpression of HSFA9 in the 35S:A9 lines temporally extended the enhancement of photomorphogenesis.

Precedent studies in transgenic plants showed that small changes in the accumulation of phytochrome proteins could cause enhanced sensitivity to light responses mediated by phytochromes (see, for example, Cherry *et al.*, 1992). Increased accumulation of phyA and phyB proteins detected in seeds and seedlings under darkness (Fig. 5B) would thus explain the enhanced responses to monochromatic light observed with the 35S:A9 seedlings under both FRc and Rc (Fig. 4). Mutant analyses in Arabidopsis suggested that *PHYA* is the only phytochrome that mediates seedling responses to FRc (see, for example, Nagatani *et al.*, 1993). In addition, *PHYB* is the main (if not the only) phytochrome involved in reduction of the hypocotyl length in response to Rc (Tepperman *et al.*, 2004). Therefore, the results in Fig. 4 prove that HSFA9 enhances light perception by *PHYA* and *PHYB*, which are perhaps the two phytochromes with the highest contribution to initial seedling greening and reduction of hypocotyl elongation. A third phytochrome, *PHYC*, has a smaller contribution to hypocotyl elongation that in Arabidopsis depends on *PHYB* (Franklin and Quail, 2010, and references therein). The moderately increased *HY5* expression observed in seeds imbibed under darkness (inferred from Fig. 5A) could also contribute to the observed enhancement of FRc- and Rc-light responses (Fig. 4). *HY5* is a high hierarchical regulator of the transcriptional cascades for photomorphogenesis that integrates signals from different photoreceptors, including *PHYA* and *PHYB*. Indeed, >60% of early FRc and Rc light-induced genes have been found to be direct HY5-binding targets (Lee *et al.*, 2007). In Arabidopsis, only *PHYA* modulates the expression of 11% of the genome; this includes a diversity of TFs involved in light-signalling cascades in imbibed seeds (Ibarra *et al.*, 2013). Therefore, HSFA9, through *PHYA* and *PHYB*, could enhance a widespread transcriptional effect after the first exposure of seedlings to light. This would impact on early photomorphogenesis by accelerating greening, as shown in Figs 2 and 3. For example, enhanced production of chlorophyllide upon illumination would contribute to the observed accelerated greening; this is a limiting step in the biosynthesis of chlorophylls that is catalysed by POR and facilitated by HSFA9 (Reinbothe *et al.*, 1999; Fig. 2A). HSFA9 also increased total carotenoid content upon seedling illumination (Fig. 2A). Carotenoids are integral accessory pigments in the light-absorbing antenna (see Rodríguez-Villalón *et al.*, 2009; reviewed by Nisar *et al.*, 2015). The observed chlorophyllide and carotenoid content increases (Fig. 2A) are consistent with HSFA9 upregulating *HY5* (Fig. 5A) and with *HY5*, in turn, activating *POR* genes and other key genes involved in carotenoid biosynthesis, as described in Arabidopsis for *PORC* and *PHYTOENE SYNTHASE* (Toledo-Ortíz *et al.*, 2014). We conclude that, in developing seeds and before the first exposure to light of seeds and seedlings, HSFA9 helps to activate gene expression relevant for photomorphogenesis, thus anticipating the light responses of seedlings. HSFA9 subsequently reinforces the initial light responses that promote early greening. Because in Asterid plants HSFA9 is placed downstream of auxin hormone signalling cascades that affect plant embryos (Carranco *et al.*, 2010), HSFA9 could integrate light (phytochrome-mediated) responses with auxin embryonic cues. Our findings do not rule out the contribution of other mechanisms; these include effects on additional light receptors or mechanisms not directly related to photomorphogenesis. HSFA9 would thus help facilitate quick seedling establishment after seedlings emerge from the soil and are exposed to light. This is a desirable and useful trait for plant crops, thus our results might open new possibilities for genetic improvement.

## Accession numbers

The SSH A9 ESTs were deposited in GenBank with accession numbers dbEST JZ897403–JZ897601 (LIBEST_028687 for the SSH A9 library).

## Supplementary Material

Supplementary Data
