# Supplementary material for: Seed-specific transcription factor HSFA9 links late embryogenesis and early photomorphogenesis
Source: J Exp Bot. 2017 Feb 16;68(5):1097–108. doi: 10.1093/jxb/erx020 (PMC5441851; doi:10.1093/jxb/erx020)
Supplement: Supplementary Data [file erx020_Supplementary_Data.zip › supplementary_figures_S1_S4_tables_S1_S3.pdf]

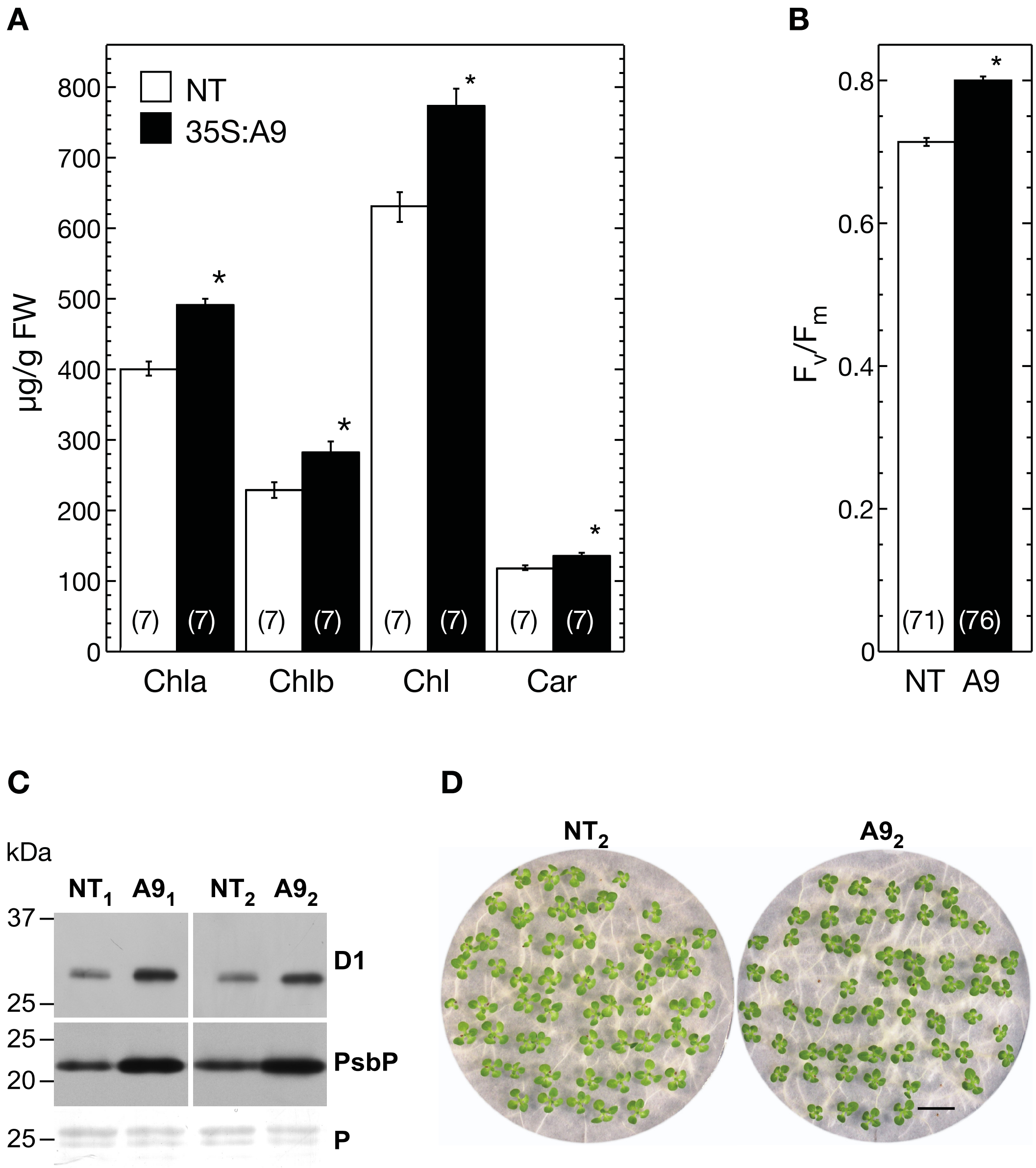

Fig. S1. Changes in the photosynthetic machinery of 35S:A9 seedlings grown under non-stress conditions.

Comparison between homozygous 35S:A9 (A9) and non-transgenic (NT) sibling lines. Experiments were performed with 2-3 different sibling line pairs, using 3-4 week-old seedlings. Methods were as described by Almoguera *et al.*, (2012).

(A) Quantification of photosynthetic pigments: chlorophyll a (Chla), chlorophyll b (Chlb), total chlorophyll (Chl), and Carotenoids (Car). Error bars denote the SEM. Numbers in brackets indicate sample size (number of Petri dishes, each dish providing average measurements from 30-60 seedlings).

(B) Maximum quantum efficiency of PSII (F_v/F_m). Error bars denote the SEM. Asterisks denote statistically significant differences: $P<0.05$. Numbers in brackets indicate sample size.

(C) Western blot showing accumulation levels of the PSII proteins PsbA (D1) and PsbP. Equal loading of total protein in samples was verified with Ponceau S staining (P).

(D) Representative photographs of 3-4 week-old seedlings depicting the NT_2 / 35S:$A9_2$ ($A9_2$) sibling pair. Scale bar = 1 cm.

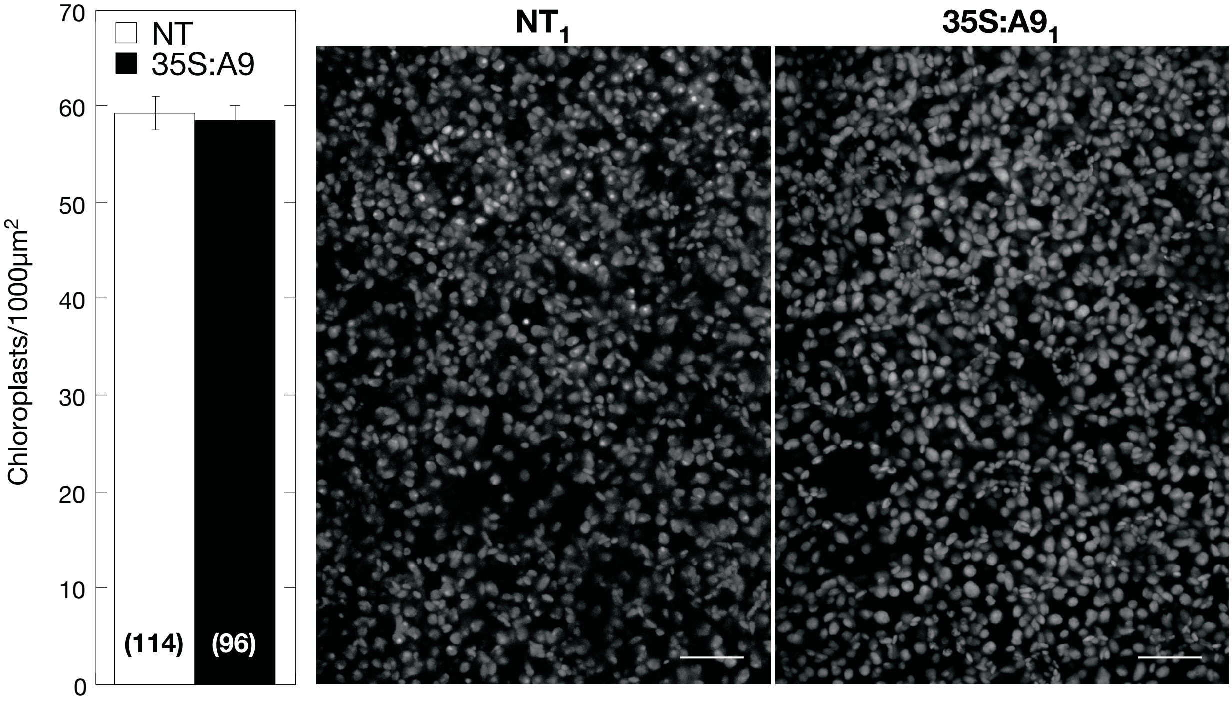

Fig. S2. Similar density of chloroplasts in cotyledons of the 35S:A9 and sibling NT seedlings.

Confocal microscopy stacked images of chloroplasts were taken as in Fig. 2, except that photo-multiplier voltage was increased to 500 V in the NT samples. This compensated for their lower intrinsic fluorescence of chlorophyll and facilitated chloroplast counting, performed after the NT and 35S:A9 images were adjusted with the required brightness and contrast changes. Average data ± SEM from 3 square fields taken from 1-2 cotyledons per plant in 5 independent experiments performed with 3 sibling line pairs is presented. Numbers in brackets denote sample size (square fields). The pictures illustrate typical chloroplast numbers in adjusted images for the NT_1/35S:$A9_1$ pair. Scale bars, 20 µm.

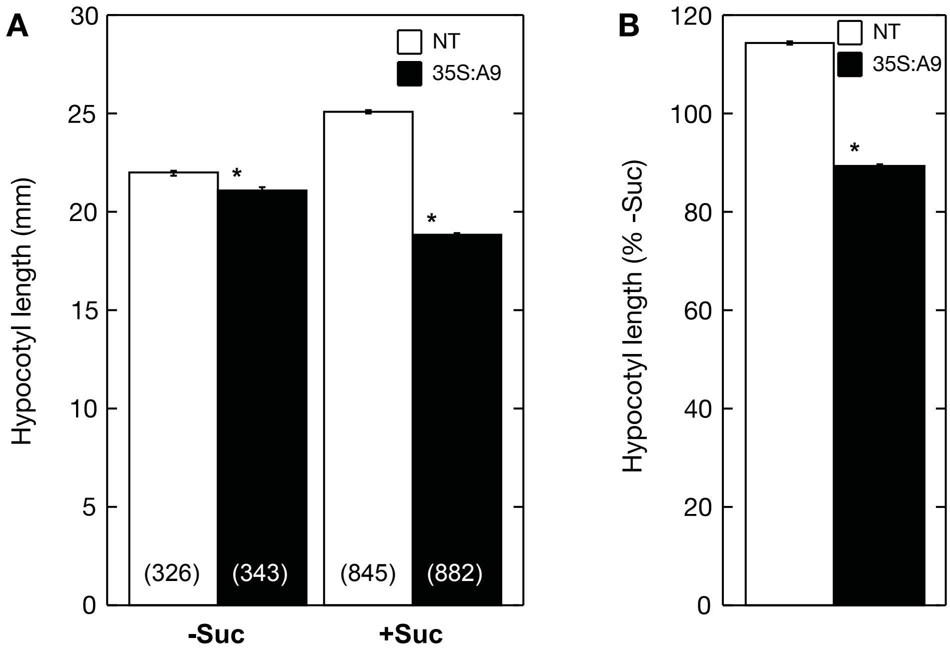

Fig. S3. Sucrose enhances the hypocotyl length reduction observed under darkness in 35S:A9 seedlings.

(A) The differences between NT and 35S:A9 seedlings observed in Fig. 4A, Dark (same data also presented here: +Suc) are compared side by side with the results of similar experiments performed using medium without added sucrose (-Suc). (B) The measured hypocotyl lengths (data from A) for seedlings in medium with sucrose are represented as the percent respect to lengths measured in medium without sucrose. A statistically significant and differential effect of sucrose was observed (see also Supplementary Table S3). Rest of symbols are as in Fig. 4.

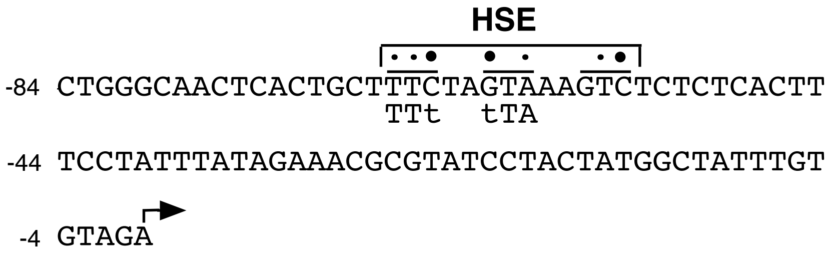

Fig. S4. Location of the imperfect HSE box in sequences immediately upstream of the *NtPHYA1* promoter. The arrow indicates the major transcription initiation site. Thin lines on the nucleotide sequence mark the TTC and GAA repeats within the HSE, with dots indicating the nucleotides that match the repeat consensus sequences. Below the WT sequence, nucleotides in lower case indicate mutations of the crucial HSF-HSE contact points (indicated by larger dots), expected to abolish HSFA9 binding (see Carranco *et al.*, 1999, and references therein).

Gene	Acc. number	Forward	Reverse
Ntubc2	AB026056	CTGGACAGCAGACTGACATC	CAGGATAATTTGCTGTAACAGATTA
L25	L18908	CCCCTCACCACAGAGTCTGC	AAGGGTGTTGTTGTCCTCAATCTT
EF-1α	AF120093	TGAGATGCACCACGAAGCTC	CCAACATTGTCACCAGGAAGTG
NtPsbA (D1)	Z00044.2	ATGGGGTCGCTTCTGTAACT	GAGCAGCAATGAAGGCAATA
PsaG	JZ897449	GGAGTGAGCCATTTTGAAGC	AGCCCCAAGCAAGAACATC
PsbR	JZ897418	TTTGTGCGGTGATGAGTTC	ATGGCTTGTCGGTCTTGAT
POR	JZ897492	GACAGGAAAATGGCATGTGA	TATCGACAAACTGGCGAACA
HSP26	JZ897404	TCTTGATGTCTCCCCTTTCG	ATTTCCCCTACTGCTGATGA
SlCOP1	XM_004250195.1	TGCTTGTATCTGGTAGTGATGATTG	GGATTATACTTCACCGAGCAAA
NtPHYA1	X66784.1	ATGTGCAAGCAGGGATGTTG	ATCCGGGTGAACTTGTCCAT
NtPHYB1	L10114.1	CTTCGCTTGGTGATGCAGTT	TGCACCACCCCACTTTATCT
SlHY5	NM_001247891.1	GATCAGAAGAGTGCCGGAGA	TTCCTTTGAGTCCCAGCTGA

Table S1. Oligonucleotide primers used for RT-qPCR.

Comparison	Statistics
35S:A9, HSP26 (Fig. 1)	$t = 2.48$, $P = 0.0306$
35S:A9, PsbR (Fig. 1)	$t = 5.49$, $P = 0.0053$
35S:A9, POR1 (Fig. 1)	$t = 2.25$, $P = 0.0461$
35S:A9, PsaG (Fig. 1)	$t = 3.19$, $P = 0.0109$
DS10:A9-SRDX, HSP26 (Fig. 1)	$t = -110.77$, $P < 0.0001$
DS10:A9-SRDX, PsbR (Fig. 1)	$t = -67.96$, $P < 0.0001$
DS10:A9-SRDX, POR1 (Fig. 1)	$t = -3.603$, $P = 0.0048$
DS10:A9-SRDX, PsaG (Fig. 1)	$t = -6.457$, $P = 0.00019$
Chlide, NT to 35S:A9, 6h under white light (Fig. 2A)	$F = 7.01$, $P = 0.0151$
Chlide, NT to 35S:A9, 16h under white light (Fig. 2A)	$F = 4.85$, $P = 0.0395$
Chl, NT to 35S:A9, 6h under white light (Fig. 2A)	$F = 12.41$, $P = 0.0014$
Chl, NT to 35S:A9, 16h under white light (Fig. 2A)	$F = 19.27$, $P = 0.00013$
Car, NT to 35S:A9, 6h under white light (Fig. 2A)	$F = 4.47$, $P = 0.042$
Car, NT to 35S:A9, 16h under white light (Fig. 2A)	$F = 16.79$, $P = 0.0002$
Chlide, NT to A9-M3, 16h under white light (Fig. 2A)	$F = 4.97$, $P = 0.0457$
Chl, NT to DS10:A9-SRDX, 6h under white light (Fig. 2A)	$F = 8.98$, $P = 0.0121$
Chl, NT to DS10:A9-SRDX, 16h under white light (Fig. 2A)	$F = 34.85$, $P < 0.0001$
Car, NT to DS10:A9-SRDX, 16h under white light (Fig. 2A)	$F = 4.51$, $P = 0.045$
Cotyledon unfolding, NT to 35S:A9 (Fig. 3)	$F = 73.92$, $P < 0.0001$
Cotyledon unfolding, NT to DS10:A9 (Fig. 3)	$F = 30.55$, $P < 0.0001$
Cotyledon unfolding, NT to DS10:A9-SRDX (Fig. 3)	$F = 11.87$, $P = 0.0015$
Hypocotyl length (mm), Dark, NT to 35S:A9 (Fig. 4A)	$F = 3300.9$, $P < 0.0001$
Hypocotyl length (mm), FRc, NT to 35S:A9 (Fig. 4A)	$F = 501.06$, $P < 0.0001$
Hypocotyl length (mm), Rc1, NT to 35S:A9 (Fig. 4A)	$F = 2258.7$, $P < 0.0001$
Hypocotyl length (mm), Rc2, NT to 35S:A9 (Fig. 4A)	$F = 2227.1$, $P < 0.0001$
Hypocotyl length (mm), FRp, NT to 35S:A9 (Fig. 4A)	$F =1352.6$, $P < 0.0001$
Hypocotyl length (%), FRc, NT to 35S:A9 (Fig. 4B)	$F = 48.261$, $P < 0.0001$
Hypocotyl length (%), Rc1, NT to 35S:A9 (Fig. 4B)	$F = 539.88$, $P < 0.0001$
Hypocotyl length (%), Rc2, NT to 35S:A9 (Fig. 4B)	$F = 543.22$, $P < 0.0001$
Hypocotyl length (%), FRp, NT to 35S:A9 (Fig. 4B)	$F = 0.263$, $P = 0.6086$
DS10:A9, COP1 (Fig. 5A)	$t = -2.422$, $P = 0.0459$
DS10:A9, PHYA (Fig. 5A)	$t = 2.988$, $P = 0.0243$
DS10:A9, PHYB (Fig. 5A)	$t = 2.344$, $P = 0.0437$
DS10:A9, HY5 (Fig. 5A)	$t = 3.315$, $P = 0.0452$
DS10:A9-SRDX, COP1 (Fig. 5A)	$t = 2.402$, $P = 0.0398$
DS10:A9-SRDX, PHYA (Fig. 5A)	$t = -14.46$, $P < 0.0001$
DS10:A9-SRDX, PHYB (Fig. 5A)	$t = -2.366$, $P = 0.0395$
DS10:A9-SRDX, HY5 (Fig. 5A)	$t = -2.775$, $P = 0.0391$
PHYA+A9 to PHYAΔ1+A9 (Fig. 6)	$F = 2.50$, $P = 0.115$
PHYAΔ2+A9 to PHYA(m)+A9 (Fig. 6)	$F = 0.865$, $P = 0.354$
Chla, NT to 35S:A9 (Fig. S1)	$F = 42.12$, $P < 0.0001$
Chlb, NT to 35S:A9 (Fig. S1)	$F = 6.75$, $P = 0.023$
Chl, NT to 35S:A9 (Fig. S1)	$F = 20.30$, $P = 0.00072$
Car, NT to 35S:A9 (Fig. S1)	$F = 5.89$, $P = 0.032$
Fv/Fm, NT to 35S:A9 (Fig. S1)	$F = 158.80$, $P < 0.0001$
Chloroplasts/1000μm^2, NT to 35S:A9 (Fig. S2)	$F = 0.114$, $P = 0.7406$
Hypocotyl lenght (mm), -Suc, NT to 35S:A9 (Fig. S3A)	$t = 4.633$, $P < 0.0001$
Hypocotyl lenght (%), NT to 35S:A9 (Fig. S3B)	$t = 49.52$, $P < 0.0001$

Table S3. Statistical data for the different comparisons mentioned in the text and Figures.

User_Id	PUT_ID	GenBank_Accn
SSH A9-1	bHLH transcription factor	JZ897403
**SSH A9-2**	**HSP26**	JZ897404
**SSH A9-3**	**HSP26**	JZ897405
**SSH A9-4**	**Aminopeptidase**	JZ897406
SSH A9-5	bHLH61	JZ897407
SSH A9-8	Nucleolar protein	JZ897408
SSH A9-9	MYB factor	JZ897409
**SSH A9-11**	**Rubisco small subunit (RbcS)**	JZ897410
SSH A9-12	LRR Kinase	JZ897411
SSH A9-13		JZ897412
SSH A9-14	Zn finger transcription factor	JZ897413
SSH A9-15		JZ897414
**SSH A9-16**	**Beta-CA**	JZ897415
SSH A9-17	Histone H3.2 like	JZ897416
SSH A9-18	protease inhibitor	JZ897417
**SSH A9-19**	**PsbR**	JZ897418
SSH A9-20	Subtilisin-like protease	JZ897419
**SSH A9-21**	**Rubisco small subunit (RbcS)**	JZ897420
SSH A9-23	Acyltransferase	JZ897421
**SSH A9-24**	**PsaH protein of PSI**	JZ897422
SSH A9-25	MIP Aquaporin	JZ897423
**SSH A9-26**	**Rubisco Activase**	JZ897424
SSH A9-27	BTB/POZ Transcription factor protein	JZ897425
SSH A9-29	Aquaporin MIP	JZ897426
**SSH A9-30**	**ABC transporter plastid**	JZ897427
SSH A9-33	Golgin candidate 5	JZ897428
SSH A9-34	Aquaporin 1	JZ897429
SSH A9-35	(phosphoenolpyruvate carboxylase) PEP carboxylase	JZ897430
SSH A9-36	Ethylen response transcription factor (ERF)	JZ897431
SSH A9-37	pit1/defensive like protein	JZ897432
SSH A9-38	Aquaporin 1	JZ897433
**SSH A9-39**	**glutamate synthase 1 [NADH], chloroplastic**	JZ897434
**SSH A9-40**	**Rubisco small subunit (RbcS)**	JZ897435
**SSH A9-41**	**Rubisco small subunit (RbcS)**	JZ897436
SSH A9-42	eIF-5A translation initiation factor, isoform 2	JZ897437
SSH A9-43	40S ribosomal protein S18	JZ897438
SSH A9-45	Pectin methyl-esterase inhibitor	JZ897439
**SSH A9-46**	**Rubisco small subunit (RbcS)**	JZ897440
SSH A9-47	H2O2 induced protein 1, MTR, cyclophilin	JZ897441
**SSH A9-48**	**PsbO1 (33kDa protein) from oxygen evolving complex of PSII**	JZ897442
SSH A9-49	Cysteine protease	JZ897443
**SSH A9-50**	**Rubisco small subunit (RbcS)**	JZ897444
SSH A9-51	Cell number regulator 6-like isoform 1	JZ897445
SSH A9-52	40S ribosomal protein S9	JZ897446
**SSH A9-53**	**PsbO1 (33kDa protein) from oxygen evolving complex of PSII**	JZ897447
SSH A9-54		JZ897448
**SSH A9-55**	**PsaG**	JZ897449
**SSH A9-56**	**Cytochrome b6f subunit 7 (PetM)**	JZ897450
**SSH A9-57**	**Oligopeptide Transporter(OPT3) Oligopeptide transporter.**	JZ897451
**SSH A9-58**	**50S Ribosomal L28 protein**	JZ897452
SSH A9-61	Receptor-like cytosolic serine/threonine-protein kinase RBK1	JZ897453
**SSH A9-62**	**Thioredoxin M**	JZ897454
**SSH A9-63**	**Ribonucleoprotein-A**	JZ897455
SSH A9-64		JZ897456
**SSH A9-65**	**Thiamin synthetase**	JZ897457
SSH A9-67		JZ897458
SSH A9-68		JZ897459

**SSH A9-70**	**Rubisco small subunit (RbcS)**	JZ897460
**SSH A9-71**	**Rubisco small subunit (RbcS)**	JZ897461
SSH A9-72	Fatty acid methyltransferase/Cyclopropane-fatty- acyl-phospholipid synthase	JZ897462
SSH A9-73		JZ897463
SSH A9-74	Lactoylglutathione lyase (Glyosalase I)	JZ897464
SSH A9-75	Polyol transporter 6-like, Sugar/carbohydrate transporter	JZ897465
**SSH A9-77**	**Chloroplastic Ribonucleoprotein B**	JZ897466
**SSH A9-78**	**Chloroplastic Ribonucleoprotein A**	JZ897467
SSH A9-79		JZ897468
SSH A9-80		JZ897469
SSH A9-81	Apoptosis-induced oxidoreductase	JZ897470
SSH A9-82	Calmodulin-related (CaM7)	JZ897471
SSH A9-83	Zn finger A20 and AN1 domain transcription factor	JZ897472
**SSH A9-84**	**Rubisco small subunit (RbcS)**	JZ897473
SSH A9-85	HaHSFA9	JZ897474
SSH A9-86	Metallothionein Type 2.	JZ897475
SSH A9-87		JZ897476
SSH A9-88	Metallothionein Type 2	JZ897477
SSH A9-89	Histone 2B.1 like	JZ897478
SSH A9-92		JZ897479
SSH A9-93	AP-4 complex subunit sigma-1	JZ897480
SSH A9-94		JZ897481
SSH A9-95	PDX-1 like protein	JZ897482
SSH A9-96	protein MIZU-KUSSEI 1-like	JZ897483
SSH A9-97	Alfa tubulin	JZ897484
SSH A9-98	Auxin repressed 12.5 kDa protein	JZ897485
**SSH A9-99**	**atpD mRNA for delta subunit of chloroplast ATP synthase**	JZ897486
SSH A9-101	SnRK1-interacting protein 1	JZ897487
SSH A9-102	Zinc finger CCCH domain-containing protein	JZ897488
SSH A9-103		JZ897489
**SSH A9-104**	**PsbO1 (33kDa protein) from oxygen evolving complex of PSII**	JZ897490
SSH A9-105	Auxin-repressed protein 12.5 kDa	JZ897491
**SSH A9-106**	**NADH:protochlorophyllide oxidoreductase 1 (POR-1)**	JZ897492
SSH A9-107	HaHSFA9	JZ897493
SSH A9-108	Histone H3.2 like	JZ897494
SSH A9-109		JZ897495
**SSH A9-110**	**Magnesium-protoporphyrin IX monomethyl ester [oxidative] cyclase.**	JZ897496
SSH A9-111		JZ897497
SSH A9-112	Inositol 2-dehydrogenase/D-chiro-inositol 3-dehydrogenase-like	JZ897498
SSH A9-114	Translationally controled tumor protein like, P23 tumor protein-like	JZ897499
SSH A9-115	ATP synthase subunit b	JZ897500
SSH A9-116	Tonoplast intrinsic protein, aquaporin TIP 1-1	JZ897501
**SSH A9-117**	**Magnesium protoporphyrin IX monomethyl ester cyclase**	JZ897502
**SSH A9-118**	**Thiamin synthetase**	JZ897503
SSH A9-119	Water channel aquaporin. MIP Aquaporin	JZ897504
**SSH A9-120**	**Beta-carbonic anhydrase (CA)**	JZ897505
**SSH A9-121**	**Chloroplast 50S Ribosomal protein L31**	JZ897506
SSH A9-122		JZ897507
**SSH A9-123**	**Rubisco small subunit (RbcS)**	JZ897508
SSH A9-124	NADH dehydrogenase [ubiquinone] 1 alpha subcomplex subunit 1-like	JZ897509
SSH A9-125	Mannosyl-oligosaccharide glucosidase	JZ897510
SSH A9-126	LRR receptor-like serine/threonine-protein kinase	JZ897511
SSH A9-127	Calcium-dependent protein kinase (CDPK) 26	JZ897512
SSH A9-128		JZ897513
SSH A9-129	Poly(A)-binding protein	JZ897601
SSH A9-130	Aux/IAA4-like transcription factor	JZ897598
**SSH A9-131**	**Nucleoside diphosphate kinase (NDPK II)**	JZ897514
**SSH A9-132**	**PsbO2 protein from the OEC of PSII**	JZ897515

SSH A9-133	Cytochrome-c reductase-processing peptidase subunit II	JZ897516
**SSH A9-135**	**NADH dehydrogenase (ubiquinone) complex I, assembly factor 6**	JZ897517
SSH A9-136	Cactin-like	JZ897599
**SSH A9-137**	**PsbX protein of PSII**	JZ897518
SSH A9-138	Melanoma-associated antigen G1 protein	JZ897519
SSH A9-139		JZ897520
SSH A9-140	PH-interacting protein-like	JZ897521
**SSH A9-141**	**CURVATURE THYLAKOID 1A (CURT1A) protein**	JZ897522
SSH A9-142	26S proteasome ATPase regulatory subunit 6	JZ897523
**SSH A9-143**	**CURVATURE THYLAKOID (CURT1) protein**	JZ897524
**SSH A9-144**	**LHCII Chlorophyll a/b-binding protein**	JZ897525
**SSH A9-145**	**Ribulose Phosphate 3-Epimerase (RPE)**	JZ897526
**SSH A9-146**	**LHCII Chlorophyll a/b-binding protein**	JZ897527
**SSH A9-147**	**Beta-Carbonic Anhydrase (CA)**	JZ897528
**SSH A9-148**	**PsaH protein of PSI**	JZ897529
**SSH A9-149**	**PsaO protein of PSI**	JZ897530
**SSH A9-150**	**PsbO2 protein from the OEC of PSII**	JZ897531
**SSH A9-151**	**FtsH-like protease**	JZ897532
SSH A9-152	AAA-ATPase. Spastin-like protein	JZ897533
**SSH A9-153**	**Tetratricopeptide repeat protein 1**	JZ897534
SSH A9-154	H+ ATP-synthetase F-type G subunit	JZ897535
**SSH A9-156**	**PsaN protein of PSI**	JZ897536
**SSH A9-157**	**Rubisco small subunit (RbcS)**	JZ897537
**SSH A9-158**	**LHCII Chlorophyll a/b-binding protein**	JZ897538
SSH A9-159	S5 protein of 40S Ribosome	JZ897539
SSH A9-160		JZ897540
SSH A9-161	CBL-interacting Ser/Threonin-Kinase	JZ897541
SSH A9-162	GUT-15 mRNA	JZ897542
**SSH A9-163**	**LHCII Chlorophyll a/b-binding protein**	JZ897543
SSH A9-164		JZ897544
SSH A9-165	Xyloglucan endoTransglucosylase- Hydrolase XTH7.	JZ897545
**SSH A9-166**	**Chlorophyll a-b binding protein 37, chloroplastic-like.**	JZ897546
SSH A9-167	40S Ribosome-associated P40 protein (SA-like)	JZ897547
SSH A9-168	Translation initiation factor 5A	JZ897548
SSH A9-169	Bidirectional sugar transporter SWEET12-like	JZ897549
SSH A9-170	Alpha-Galactosidase 1 like	JZ897550
SSH A9-171	Serine-Carboxypeptidase-like 11	JZ897551
**SSH A9-172**	**Fructose 1,6 bisphosphate Aldolase**	JZ897552
SSH A9-173	SNF1-related protein kinase regulatory subunit beta-1	JZ897553
SSH A9-174	bHLH transcription factor	JZ897554
SSH A9-175	NADP-Glutamate deshydrogenase	JZ897555
**SSH A9-176**	**LHCII Chlorophyll a/b-binding protein**	JZ897556
**SSH A9-177**	**UK-114 ribonuclease-like.**	JZ897557
**SSH A9-178**	**LHCII Chlorophyll a/b-binding protein**	JZ897558
SSH A9-179		JZ897559
SSH A9-180	CLIP-associating protein	JZ897560
SSH A9-182	Auxin repressed 12.5 kDa protein.	JZ897561
SSH A9-183	Nuclear cap-binding protein subunit 1-like	JZ897562
SSH A9-184	Profilin 2-like	JZ897563
**SSH A9-185**	**LHCII Chlorophyll a/b-binding protein**	JZ897564
SSH A9-186	EKC/KEOPS complex subunit bud32-like	JZ897565
**SSH A9-187**	**Rubredoxin-like**	JZ897566
**SSH A9-188**	**Chloroplastic group IIA intron splicing facilitator CRS1**	JZ897567
**SSH A9-189**	**LHCII Chlorophyll a/b-binding protein**	JZ897568
SSH A9-191	Pseudouridine 5' monophosphatase-like	JZ897569
SSH A9-192	IAA27	JZ897570
SSH A9-193	Protein with YTH domain	JZ897571
SSH A9-194	Bifunctional nuclease 2-like	JZ897572

**SSH A9-195**	**Triose-phosphate isomerase, chloroplastic-like**	JZ897573
SSH A9-196		JZ897574
**SSH A9-197**	**Chloroplast pigment-binding protein CP29**	JZ897575
SSH A9-199	Aux/IAA4-like Transcription Factor	JZ897576
**SSH A9-200**	**LHCII Chlorophyll a/b-binding protein**	JZ897577
SSH A9-201		JZ897578
SSH A9-202	Aquaporin TIP1	JZ897579
SSH A9-203		JZ897580
**SSH A9-204**	**Protease Do-like 1, chloroplastic-like**	JZ897581
SSH A9-205	Peptidyl-Prolyl cis-trans isomerase CYP23-like	JZ897582
SSH A9-206		JZ897583
SSH A9-207	Profilin 2-like	JZ897584
**SSH A9-208**	**PsbP protein. PSII OEC protein.**	JZ897585
SSH A9-209	60S Ribosomal L16 protein	JZ897586
SSH A9-210	Ethylen-Insentive-like transcription factor (EIL)	JZ897587
**SSH A9-211**	**Rubisco small subunit (RbcS)**	JZ897588
**SSH A9-212**	**Rubisco small subunit (RbcS)**	JZ897600
**SSH A9-213**	**Aspartate aminotrasferase**	JZ897589
SSH A9-214		JZ897590
SSH A9-215	RINT1-like protein-like	JZ897591
SSH A9-216	Dephospho-CoA kinase domain-containing protein-like	JZ897592
SSH A9-217	Repetitive proline-rich cell wall protein 1	JZ897593
**SSH A9-218**	**Adenylyl-sulfate kinase 3, chloroplastic-like**	JZ897594
SSH A9-219	Basic beta-1,3-glucanase	JZ897595
**SSH A9-220**	**HSP26**	JZ897596
SSH A9-221	60S ribosomal protein L18a-2-like	JZ897597

Table S2. List of the SSH-A9 cloned cDNAs.
We include the putative identity (ID) and the accession number corresponding to each clone.
In bold face (and outlined in green color), we indicate the cDNAs encoding products with potential functions connected with the structure and assembly of the photosynthetic apparatus, and (or) with predicted chloroplast localization.

Comparison	Statistics
35S:A9, HSP26 (Fig. 1)	$t = 2.48$, $P = 0.0306$
35S:A9, PsbR (Fig. 1)	$t = 5.49$, $P = 0.0053$
35S:A9, POR1 (Fig. 1)	$t = 2.25$, $P = 0.0461$
35S:A9, PsaG (Fig. 1)	$t = 3.19$, $P = 0.0109$
DS10:A9-SRDX, HSP26 (Fig. 1)	$t = -110.77$, $P < 0.0001$
DS10:A9-SRDX, PsbR (Fig. 1)	$t = -67.96$, $P < 0.0001$
DS10:A9-SRDX, POR1 (Fig. 1)	$t = -3.603$, $P = 0.0048$
DS10:A9-SRDX, PsaG (Fig. 1)	$t = -6.457$, $P = 0.00019$
Chlide, NT to 35S:A9, 6h under white light (Fig. 2A)	$F = 7.01$, $P = 0.0151$
Chlide, NT to 35S:A9, 16h under white light (Fig. 2A)	$F = 4.85$, $P = 0.0395$
Chl, NT to 35S:A9, 6h under white light (Fig. 2A)	$F = 12.41$, $P = 0.0014$
Chl, NT to 35S:A9, 16h under white light (Fig. 2A)	$F = 19.27$, $P = 0.00013$
Car, NT to 35S:A9, 6h under white light (Fig. 2A)	$F = 4.47$, $P = 0.042$
Car, NT to 35S:A9, 16h under white light (Fig. 2A)	$F = 16.79$, $P = 0.0002$
Chlide, NT to A9-M3, 16h under white light (Fig. 2A)	$F = 4.97$, $P = 0.0457$
Chl, NT to DS10:A9-SRDX, 6h under white light (Fig. 2A)	$F = 8.98$, $P = 0.0121$
Chl, NT to DS10:A9-SRDX, 16h under white light (Fig. 2A)	$F = 34.85$, $P < 0.0001$
Car, NT to DS10:A9-SRDX, 16h under white light (Fig. 2A)	$F = 4.51$, $P = 0.045$
Cotyledon unfolding, NT to 35S:A9 (Fig. 3A)	$F = 73.92$, $P < 0.0001$
Cotyledon unfolding, NT to DS10:A9 (Fig. 3A)	$F = 30.55$, $P < 0.0001$
Cotyledon unfolding, NT to DS10:A9-SRDX (Fig. 3A)	$F = 11.87$, $P = 0.0015$
Hypocotyl length (mm), Dark, NT to 35S:A9 (Fig. 4A)	$F = 3300.9$, $P < 0.0001$
Hypocotyl length (mm), FRc, NT to 35S:A9 (Fig. 4A)	$F = 501.06$, $P < 0.0001$
Hypocotyl length (mm), Rc1, NT to 35S:A9 (Fig. 4A)	$F = 2258.7$, $P < 0.0001$
Hypocotyl length (mm), Rc2, NT to 35S:A9 (Fig. 4A)	$F = 2227.1$, $P < 0.0001$
Hypocotyl length (mm), FRp, NT to 35S:A9 (Fig. 4A)	$F = 1352.6$, $P < 0.0001$
Hypocotyl length (%), FRc, NT to 35S:A9 (Fig. 4B)	$F = 48.261$, $P < 0.0001$
Hypocotyl length (%), Rc1, NT to 35S:A9 (Fig. 4B)	$F = 539.88$, $P < 0.0001$
Hypocotyl length (%), Rc2, NT to 35S:A9 (Fig. 4B)	$F = 543.22$, $P < 0.0001$
Hypocotyl length (%), FRp, NT to 35S:A9 (Fig. 4B)	$F = 0.263$, $P = 0.6086$
DS10:A9, COP1 (Fig. 5A)	$t = -2.422$, $P = 0.0459$
DS10:A9, PHYA (Fig. 5A)	$t = 2.988$, $P = 0.0243$
DS10:A9, PHYB (Fig. 5A)	$t = 2.344$, $P = 0.0437$
DS10:A9, HY5 (Fig. 5A)	$t = 3.315$, $P = 0.0452$
DS10:A9-SRDX, COP1 (Fig. 5A)	$t = 2.402$, $P = 0.0398$
DS10:A9-SRDX, PHYA (Fig. 5A)	$t = -14.46$, $P < 0.0001$
DS10:A9-SRDX, PHYB (Fig. 5A)	$t = -2.366$, $P = 0.0395$
DS10:A9-SRDX, HY5 (Fig. 5A)	$t = -2.775$, $P = 0.0391$
PHYA+A9 to PHYAΔ1+A9 (Fig. 6)	$F = 2.50$, $P = 0.115$
PHYAΔ2+A9 to PHYA(m)+A9 (Fig. 6)	$F = 0.865$, $P = 0.354$
Chla, NT to 35S:A9 (Fig. S1)	$F = 42.12$, $P < 0.0001$
Chlb, NT to 35S:A9 (Fig. S1)	$F = 6.75$, $P = 0.023$
Chl, NT to 35S:A9 (Fig. S1)	$F = 20.30$, $P = 0.00072$
Car, NT to 35S:A9 (Fig. S1)	$F = 5.89$, $P = 0.032$
Fv/Fm, NT to 35S:A9 (Fig. S1)	$F = 158.80$, $P < 0.0001$
Chloroplasts/1000μm^2, NT to 35S:A9 (Fig. S2)	$F = 0.114$, $P = 0.7406$
Hypocotyl length (mm), -Suc, NT to 35S:A9 (Fig. S3A)	$t = 4.633$, $P < 0.0001$
Hypocotyl length (%), NT to 35S:A9 (Fig. S3B)	$t = 49.52$, $P < 0.0001$

Table S3. Statistical data for the different comparisons mentioned in the text and Figures.
